# Industrial hemp seed: from the field to value-added food ingredients

**DOI:** 10.1186/s42238-022-00156-7

**Published:** 2022-07-29

**Authors:** Rachel A. Burton, Mike Andres, Martin Cole, James M. Cowley, Mary Ann Augustin

**Affiliations:** 1 Department of Food Science, School of Agriculture, Food and Wine, University of Adelaide, Waite Campus, Urrbrae, SA 5064 Australia; 2 Plant Genomics Centre, Waite Campus Receivals, Corner of Hartley Grove and Paratoo Road, Urrbrae, SA 5064 Australia; 3 CSIRO Business Development & Global, CSIRO Building 122, Research Way, Clayton, VIC 3168 Australia; 4 Present Address: Wine Australia, Industry House Corner Hackney and Botanic Roads, Adelaide, SA 5000 Australia; 5 CSIRO Agriculture & Food, 671 Sneydes Road, Werribee, VIC 3030 Australia

**Keywords:** *Cannabis*, Market, Agronomy, Hemp seed, Food ingredients

## Abstract

Industrial hemp, with low levels of the intoxicating cannabinoid tetrahydrocannabinol (THC), is grown for fibre and seeds. The industrial hemp industry is poised for expansion. The legalisation of industrial hemp as an agricultural commodity and the inclusion of hemp seed in foods is helping to drive the expansion of the hemp food ingredients industry. This paper discusses the opportunity to build an industrial hemp industry, with a focus on the prospects of hemp seed and its components in food applications. The market opportunities for industrial hemp products are examined. Various aspects of the science that underpins the development of an industrial hemp industry through the food supply chain are presented. This includes a discussion on the agronomy, on-farm and post-harvest considerations and the various types of food ingredients that can be made from hemp seed. The characteristics of hemp seed meal, hemp seed protein and hemp seed oil are reviewed. Different processes for production of value-added ingredients from hemp seed, hemp seed oil and hemp seed protein, are examined. The applicability of hemp seed ingredients in food applications is reviewed. The design of hemp seed ingredients that are fit-for-purpose for target food applications, through the selection of varieties and processing methods for production of various hemp seed ingredients, needs to consider market-led opportunities. This will require an integrated through chain approach, combined with the development of on-farm and post-farm strategies, to ensure that the hemp seed ingredients and foods containing hemp seed are acceptable to the consumer.

## Background

*Cannabis sativa,* native to Eastern Asia, was probably first cultivated in China (*circa* 2700 BC) for its medicinal properties. It is a potentially profitable, multi-use crop that has good sustainability credentials due to its low environmental impact (Ranalli and Venturi 2004). Hemp seed is highly nutritious, containing around 25% protein, 35% oil, and high levels of antioxidants (Callaway 2004; Farinon et al. 2020). The vegetative biomass from hemp has applications as building materials and in biofuel production, and its stem fibres can be used to produce textiles (Farinon et al. 2020). Hemp has deep tap roots which enables efficient water acquisition and the storage of carbon and which are also effective at improving soil structure (Farinon et al. 2020).

Of particular importance is the wide range of phytochemicals found in hemp inflorescences that have wide applications in food, pharmaceutical and cosmetic industries. The two major and better-known phytochemicals in hemp are cannabinoids, (–)-*trans*-Δ^9^-tetrahydrocannabinol (THC) which has an intoxicating effect, and (-)-cannabidiol (CBD), which is non-intoxicating but has potential medicinal benefits. As such, CBD supplements are currently highly sought after by the nutritional supplement industry (Cerino et al. 2021).

Industrial hemp—*Cannabis* varieties with low tetrahydrocannabinol (THC) levels—are grown primarily for the production of fibre and/or seeds. The impetus for the budding global industrial hemp industry is the result of key changes to legislation, namely the 2014 and 2018 Farm Bills in the USA. The 2014 Farm Bill defined hemp as plants with a THC threshold of 0.3% or less on a dry weight basis (compared with *c.* 15% for illicit drug-type *Cannabis* (ElSohly et al. 2021)) and allowed its production under specific conditions. The 2018 Farm Bill built on this by legalising the production of hemp as an agricultural commodity and removed it from the list of controlled substances (American Farm Bureau Federation 2019). Australia legalised hemp foods in November 2017 after a ban that had been in place since 1937 (The Farmer Magazine 2020).

The aim of this paper is to review the opportunity for designing a whole supply chain for industrial hemp, with a focus on the prospects of hemp seed for the food industry. For decades, hemp seed has been an under-valued co-product from industrial hemp production, primarily grown for its fibre. Subsequently, most varieties have not been optimised for the production of seed or high-quality oil and protein. Industrial hemp is an exciting alternative in a landscape where we traditionally grow a limited number of crops, where it provides an opportunity to widen our agribiodiversity. The potential of industrial hemp seed remains underdeveloped and so to highlight its promise and emerging role as a valuable source of protein and oil for the food industry, in this review, we will cover the market opportunities for industrial hemp products, discuss the growth, harvesting and storage requirements of industrial hemp seed, and describe the nutritional attributes of hemp seed and the processing/fractionation it requires to produce value-added food ingredients. Finally, recommendations will be made for future research which we anticipate will enable the development of sustainable industrial hemp supply chains, stimulating a call to action for the industrial hemp industry.

## Market opportunity

Globally, it is estimated that at least 47 countries grow hemp for commercial or research purposes (Cannabis Business Plan 2021). In 2019, Brightfield Group estimated that the top 10 producing nations alone had 864,000 acres of industrial hemp under cultivation (S&P Global Market Intelligence 2019). However, as of September 2021, there are no official estimates available that reliably and comprehensively track worldwide industrial hemp under cultivation. FAO Statistics, for example, do not capture five of the top 10 producing nations, even though Canadian figures, which are not captured by FAO, are available directly from the Canadian Government (FAOSTAT 2020; Government of Canada 2020). Due to the recency of legislative changes, the Australian industrial hemp industry is still in its infancy, with an estimated 6180 acres under cultivation, of which 64% are in Tasmania (Gordon and Brodrick 2020).

The global market size estimates for industrial hemp for 2025 vary greatly, with some estimates as high as USD 26.6 billion (MarketsandMarkets 2019). There are three key reasons for this. Firstly, the lack of official estimates for hemp under cultivation severely limits forecasting. Secondly, the crash in wholesale prices for nearly all hemp products in the USA during the second half of 2019 led to around 60% of growers struggling to find buyers due to massive oversupply (ACS Laboratory 2020). During this time prices for hemp seeds and crude hemp oil fell 18% and 68% respectively (ACS Laboratory 2020). Thirdly, there are thousands of applications for hemp, with food and beverage, fibre (textiles and paper), and beauty and personal care standing out as the three largest market opportunities (Whitney Economics 2019). Given that each of these opportunities plays into a global billion-dollar market, with 2019 market size estimates for packaged food and beverage at USD 4837 billion (Euromonitor International 2020a), textiles at USD 1587 billion (Technavio 2019), and beauty and personal care at USD 503 billion (Euromonitor International 2020b), even a small change in the size of the estimated captured market can lead to huge differences in market size estimates. Given the still nascent state of the global industrial hemp market, more conservative market size estimates, such as those from Magna Intelligence seem more reliable. Magna expects the global industrial hemp market to grow from USD 4.6 billion in 2019 to USD 9.4 billion by 2025, at an annual growth rate of 12.8% (Magna Intelligence 2020).

Although hemp can be grown as a dual-purpose crop, production systems are usually geared to producing either fibre or seed, and generally not both (AgriFutures Australia 2017). While a production system geared towards seed will not yield the long bast fibres desired in textile production, it will still yield some shorter fibres (bast and hurd or pulp) after seed harvesting, that could be used in hemp paper production (Industrial Hemp Western Australia Association Inc 2021). Hence, a seed production operation could potentially play into three key segments of the global industrial hemp market—food and beverage, personal care products, and paper. Together, these three segments represent a USD 5.1 billion opportunity comprising 53.9% of the global industrial hemp market by 2025 (Magna Intelligence 2020).

Aside from regulatory hurdles, there are currently two main obstacles that hold back the industrial hemp industry. The first is the risk of a hemp crop “going hot” (exceeding the legislated THC limit), requiring the entire crop to be destroyed. Current cultivars, even when grown successfully in previous years, may produce THC levels above the legal threshold in the following year due to varying growing conditions (ABC News 2021). The second challenge is the lack of proper planting and harvesting equipment. For example, current air-seeders used in planting can only be operated with low air volumes due to the fragility of hemp seeds (MarketsandMarkets 2019). Machinery currently used in the hemp harvest, especially smaller equipment such as sickle-bar mowers and hay swathers, struggle with frequent clogging and blunting of blades (AgriFutures Australia 2017). As planting increases, mechanised planting and harvesting needs to be refined for efficiency alongside commercial-scale processing facilities.

Despite these challenges, the plant-based protein products market is ripe for new protein sources. The two main protein sources in 2020 were soy and wheat, contributing 57.6% and 36.8% to the overall market, respectively (Technavio 2021). However, both soy and wheat are among the top eight major food allergens, which account for 90% of all food allergy reactions (Messina and Venter 2020). Wheat is also problematic for about 109 million people worldwide, or 1.4% of the global population in 2020, who suffer from coeliac disease (Singh et al. 2018; United Nations 2019). Furthermore, while meat consumption is extensive around the globe (an average of 86% of people in 39 countries surveyed saying that their diet includes meat (Statista 2020)), consumer health concerns and the desire for more sustainable and ethical products is driving the global demand for non-meat protein alternatives such as plant-based meats and dairy (Euromonitor International 2020c). Many consumers are continuing to move towards flexitarian or plant-based diets, with 23% of all consumers indicating that they try to limit their intake of meat (Euromonitor International 2020c). Novel plant-protein sources such as hemp, not known to cause food allergies or affecting coeliac sufferers, therefore are well-positioned to meet the growing global demand for plant-based proteins.

## Industrial hemp—cultivation, harvesting and storage

### Growing

Hemp is a fast-growing productive crop that is regarded as sustainable, despite the fact that in many areas of the world it requires irrigation (Tang et al. 2018). For a productive crop, hemp might require more water than dryland cereals (Schultz et al. 2020), but substantially less than other fibre crops such as cotton (Andre et al. 2016). There is still a lack of accurate studies into best practice water use (Adesina et al. 2020; Amaducci et al. 2000) but a recent study reported that hemp can survive and reproduce under extreme water deficit (Gill et al. 2022). Under ideal conditions hemp can grow very rapidly and at high sowing rates will achieve canopy closure quickly, suppressing weed growth (Struik et al. 2000). It has been shown to have a positive effect on the soil and can also be used for phytoremediation, particularly for the removal of heavy metals such as lead (Burczyk et al. 2008). Although frequently touted as requiring low inputs, hemp requires large amounts of nitrogen input for good plant establishment and there are few studies into the impact that plant nutrition has on subsequent seed and fibre quality (Herppich et al. 2020). There is sparse information on salinity tolerance of hemp across all growth stages (Cheng et al. 2016; Hu et al. 2018). Given that a successful hemp crop is dependent on irrigation, and that irrigation water and soils are becoming increasingly salinised around the world (Brady et al. 2008), hemp’s salinity tolerance will be important to define and incorporate into breeding programs.

There are established hemp breeding programs for fibre and seed with many of these in the commercial sector, with a general lack of knowledge across the industry about the type of germplasm available. There is limited information about the availability of elite breeding lines for improved seed size and quality (Small 2018). Quality factors of hemp seed including total oil content, fatty acid ratios, total protein content and beneficial phytochemicals are known to be highly variable among genotypes, as well as contents of anti-nutrients like phytate (Schultz et al. 2020). There is a strong need for a core germplasm resource to be set up for industrial hemp to support genomic efforts to unravel many key traits including those related to seed (Small 2018; Hurgobin et al. 2021; Welling et al. 2016). It is likely that a wide range of cultivars will be needed to meet the needs of different growing regions, not just around the world, but even within the same country (Schluttenhofer and Yuan 2017). Australia is a prime example of this since the northern states have hot, wet summers and cooler, drier winters ideal for hemp cultivation, however the day length requirements of hemp to trigger flowering are not ideal at these latitudes. In contrast, the southern states grow industrial hemp in the dry, hot summer months where the crop is dependent on irrigation but flowering can be more confidently predicted. Cultivars that can push the seasonal envelope, establishing well in the colder and wetter spring months of southern Australia and thus need far less irrigation, will further extend the sustainability credentials of this crop.

### Harvesting

There is a huge range of phenotypic variety displayed by the hemp cultivars currently available. This bodes well for future breeding efforts and is further augmented by the large amount of genetic variation present in germplasm collections that are not well described and therefore remain relatively untouched to date (Welling et al. 2016). There will need to be synergy between the crop types grown for seed and the development of efficient harvesters. Fibre crops are tall and densely planted and will require different harvesting specifications to shorter and bushier seed crops, planted at lower density (Huang et al. 2017). Where crops are grown for dual purpose, with the seed heads harvested first and then the remaining stalks collected for fibre, harvesting considerations will be more complex. Efficient separation of seed from biomass during on-field harvesting is ideal and potentially this can be achieved by the use of modified cereal combines (Pahole et al. 2018). However, there are currently only a few bespoke hemp harvesters available for commercial use. The HempTrain^TM^ is an advance on traditional post-harvest processing methods for decortication and separation and production of high value products. It enables separation of baled hemp straw and may be used for processing of fresh/green feedstock and dry feedstock (Canadian Greenfield Technologies Corp 2021).

The timing of harvest is also likely to have an impact on seed quality, particularly with regard to the lipid profiles. The content of linoleic, α-linolenic, oleic, and palmitic fatty acids varied with harvesting times, as did the polyunsaturated to saturated ratio, and could be correlated with seed ripeness (Marzocchi and Caboni 2020). A recent survey of hemp cultivars grown in Italy demonstrates that multiple properties, including phenolic and sterolic contents of hemp seeds, can be affected by both terroir and harvest method (Calzolari et al. 2021). Lastly, there is little known about the influence of harvesting times, and storage, on the anti-nutritional contents of hemp seeds, including phytate and lignanamide (Mattila et al. 2018) which will also influence downstream food uses.

### Postharvest segregation and storage

Both post-harvest segregation and correct storage of hemp seed will be essential. The high oil content of hemp seed means that seed quality is likely to be influenced by storage conditions, in turn dictating downstream use that is impacted by oil oxidation and rancidity (Suriyong et al. 2015). The protective compounds in hemp seeds which can help to stabilise the lipid profile during storage, can vary widely by cultivar, and thus shelf life will also vary (Izzo et al. 2020). A better understanding of factors that delay or prevent rancidity will be essential to ensure a supply of consistently high-quality seed. The effects of storage regimes on hemp seed protein profiles are little known and given that hemp protein is likely to become an important alternative plant protein resource (Shen et al. 2021), this will need further attention.

Segregation of seed types during storage and downstream processing will also be important to maintain the status of hemp seed as a credible gluten-free ingredient. Contamination from common cereals such as wheat and barley, for example via the use of shared harvesters and/or storage facilities, could occur, as has been seen for other gluten-free grains such as buckwheat (Atasoy et al. 2020).

## Food ingredients from hemp seed

**Fig. 1:**
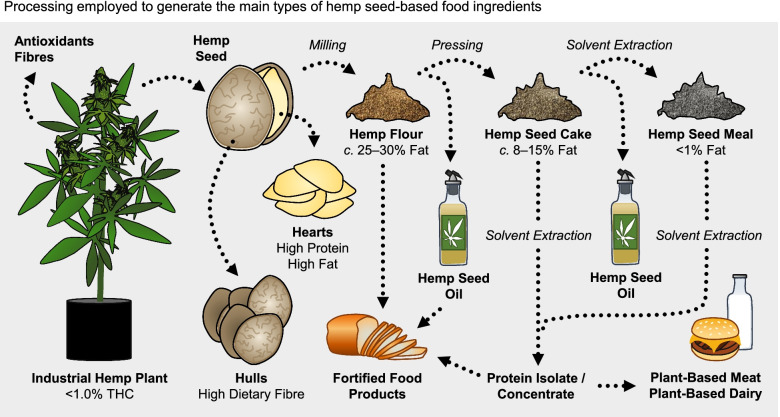
Food-grade hemp seed is harvested from industrial hemp plants with less than 1% (–)-*trans*-Δ^9^-tetrahydrocannabinol (THC). The hemp seed is comprised of an outer hull which is high in dietary fibre and the heart (seed embryo) which is rich in protein and fats. Whole hemp seeds are milled into hemp flour which is sequentially extracted to produce hemp oil and hemp protein concentrates/isolates which are used for food formulation. Residues from these extractions, hemp seed cake and hemp seed meal, can also be used to fortify foods with antioxidants and dietary fibre. Residue hemp plant biomass may also be a source of antioxidant compounds and short fibres for materials.

There is a range of hemp seed products in the market including whole hemp seed and dehulled hemp seed, hemp seed oil, hemp seed cake (the by-product after mechanical oil pressing), hemp seed meal (the by-product after solvent-based protein extraction of pressed cake), hemp hulls and hemp protein concentrates/isolates (Fig. 1). Hemp seed oleosomes, which are in-tact oil bodies, are an emerging ingredient that may be isolated from hemp seed.

Knowing the composition, nutritional properties and techno-functionality of hemp-based food ingredients is of fundamental importance for determining their fitness-for-purpose in formulated food. In addition, understanding of the manufacturing processes for hemp seed-based ingredients is also relevant as the composition, quality, and functional properties of the ingredient are influenced by the post-harvest handling and processing. The properties of the ingredients are also influenced by the cultivar, seasonality and growing conditions of the plant.

### Composition of hemp seed

The variety and agronomic conditions affect the composition of the hemp seed as detailed in Section 3 above and composition of major and minor components show significant variation. Hemp seed is a good source of macronutrients (proteins, fat and fibre), minerals (P, K, Mg, Ca, Na, Fe, Mn, Zn, Cu) and phytonutrients (tocopherols, carotenoids, sterols) but also contains a number of anti-nutritional components (phytic acid, condensed tannins, trypsin inhibitors, cyanogenic glycosides and saponins) (Callaway 2004; Leonard et al. 2020).

### Whole hemp seed, dehulled hemp seed and hulls

Typically, whole hemp seeds contain 25–35% fat, 20–25% protein, and 20–30% carbohydrates which is mainly insoluble dietary fibre. However, the variability reported is highly dependent on cultivar and agronomic conditions (Farinon et al. 2020). Hemp seed sampled from commercial sources were found to be comprised of 90.8–96.0% dry matter, 25.4–33.0% fat, 21.3–27.3% protein, 21.8–26.1% acid detergent fibre, 27.8–36.2% neutral detergent fibre and 3.7–5.9% ash (House et al. 2010). An Italian study of 20 non-drug hemp cultivars and wild hemp accessions reported oil content to range from 28.5–36% dry matter for oil and protein ranging from 31.6–35.6% dry matter(Galasso et al. 2016). The seed composition of ten industrial hemp cultivars (Alyssa, Anka, CanMa, CFX1, CFX2, CRS1, Delores, Finola, Jutta, Yvonne) grown in Canada were reported to be 26.9–30.6% oil, 23.8–28.0% protein, 25.9–29.8% acid detergent fibre, 32.7–38.8% neutral detergent fibre and 5.1–5.8% ash (Vonapartis et al. 2015). A compositional analysis of seven hemp cultivars (Bialobrzeskie, Felina 32, Tygra, Futura 75, Santhica 27, Fedora 17, Finola) grown in Greece over 3 years indicated that contents ranged from 8.5-29.2% for oil, 12.2-25.4% for protein, 40.8–74.5% for carbohydrates and 4.4-5.3% ash, with cv. Finola having the highest protein and oil content and lowest carbohydrate content (Irakli et al. 2019).

A comparison of the composition of dehulled hemp seed and hulls indicates how the individual components are distributed in the fractions. This would also be dependent on the method used in the dehulling operations. An analysis of six commercial dehulled hemp seed samples found that they contained 93.7–97.0% dry matter, 37.6–52.3% fat, 30.3–38.7% protein, 0.6–12.0% acid detergent fibre, 4.6–18.1% neutral detergent fibre and 5.4–7.1% ash (House et al. 2010). By contrast, the hulls of three commercial samples contained (on an as is basis) 93.4–97.0% dry matter, 4.3–15.8% fat, 8.8–16.3% protein, 44.9–56.9% acid detergent fibre, 55.7–74.2 % neutral detergent fibre and 3.1–4.4% ash (House et al. 2010). Distribution of components between the heart and hull reported by House et al. (House et al. 2010) are consistent with a recent microscopic study which found high levels of xylan, an important human dietary fibre, located within the hull, while the embryo contained numerous protein bodies (Schultz et al. 2020).

### Hemp seed oil

Hemp seed oil is a rich source of polyunsaturated fatty acids (> 70%), particularly the essential fatty acids linoleic acid (18:2 n-6) and α-linolenic acid (18:3 n-3) with a good n-6 to n-3 ratio (2.5–5.5) for human health. Various levels of linoleic acid (51.6–59.0% fatty acids in oil), α-linolenic acid (10.5–22.0% fatty acids in oil), total tocopherols (56–97 mg/100 g of oil), phytosterols (279 mg/100 g of oil) have been reported showing the wide variability in oil composition (Callaway 2004; Farinon et al. 2020; Irakli et al. 2019). Virgin hemp seed oil produced by screw-pressing only is a green colour because of the chlorophyll co-extracted with the oil (Matthäus and Brühl 2008).

### Hemp seed protein

Hemp seeds have protein content in a similar range to that of other oilseeds. They have a higher protein level than sunflower seeds or canola seeds but are lower in protein compared to soybean (Potin and Saurel 2020). Hemp seed proteins contain all the essential amino acids for human health and the protein fraction of hemp is highly digestible (Farinon et al. 2020; Mattila et al. 2018; House et al. 2010).

Globulins account for 60–80% of the total protein while albumins constitute the majority of the rest. Hemp globulins are mainly comprised of the 11S edestins, which is a hexameric legumin with a MW of ~ 320 kDa. Each monomer of the hexamer is ~ 54 kDa and composed of an acid α subunit and a heterogenous basic β subunit. The minority proteins in the globulin fraction are 7S globulins consisting of a subunit of ~ 48 kDa. The albumin fraction has a less compact structure than the globulin fraction and comprises seven polypeptides of 6–35 kDa. The protein fraction also contains other minor proteins (Potin and Saurel 2020; Pihlanto et al. 2020; Tang et al. 2006).

### Minor components

Hemp seeds contain a range of phytochemicals, with the presence of phenolic compounds and tocopherols contributing to their antioxidant activity. Agronomic conditions can affect the levels of phenolic compounds in the hemp seed. For example, growing hemp seed at high density without pre-seeding fertilisation led to an accumulation of phenolic compounds; a typical stress response (Faugno et al. 2019). Hemp seeds also contain some antinutritional factors like trypsin inhibitors that affect protein digestibility and bioavailability, along with phytate/phytic acid that chelates minerals like iron and zinc, reducing their bioavailability. In one study, the concentration of phytic acid in hemp seed meal was found to range from 43.8–75.5 g/kg dry matter, while trypsin inhibitors ranged from 10.8 to 27.8 unit/mg (Galasso et al. 2016). Levels of these anti-nutrients are comparable or lower than other oilseeds (Galasso et al. 2016). Condensed tannins, cyanogenic glycosides and saponins are present at levels of 1.75, 0.23 and 69 mg/kg dry matter respectively (Russo and Reggiani 2013). Further details on the mineral composition, unsaponifiable components (e.g. tocopherols, phytosterols, carotenoids) and anti-nutritional factors and their effects on nutritional quality of hemp ingredients are available in several reviews (Callaway 2004; Farinon et al. 2020; House et al. 2010; Galasso et al. 2016; Vonapartis et al. 2015).

Cannabinoids are reported to exist in very low levels in hemp seed (Jang et al. 2020) even in seed from drug-type varieties where cannabinoid contents of flower tissues can be very high (Ross et al. 2000). One study has reported that cannabinoid contents in hemp seed might be underestimated by typical analyses and, in some instances, might exceed ten times the legal limit by weight (Yang et al. 2017). As cannabinoid synthesis occurs in glandular trichomes not present in seeds, this discrepancy in content may be due to differences in contamination from vegetative materials. Further work should be done to determine if cannabinoid contents of hemp seeds are indeed this variable or a function of contamination.

## Processing of value-added hemp seed ingredients

There is a range of products that can be derived from hemp seed. Apart from the milling of whole or dehulled hemp seed into flour, value-added oil- and protein-enriched fractions may be isolated. Additionally, phytochemical extracts are becoming of increasing interest as their role and application in health and nutrition are increasingly being recognised (Rupasinghe et al. 2020).

### Oil extraction

Hemp seed oil may be extracted from whole or dehulled hemp seed (House et al. 2010). Conventional processing techniques are primarily aimed at extracting oil efficiently and to obtain an oil of good quality. Opportunities for improvements in processing for hemp seed and production of fractions from hemp seed oil will be similar to that for the processing of more established oil seeds such as soybean, rapeseed/canola seed, sunflower seed, safflower seeds, flax seeds and palm kernels. The various methods for extraction of oil from oilseeds that have been applied to hemp seed include mechanical pressing, solvent extraction, use of supercritical CO_2_, and microwave or ultrasound assisted processing (Devi and Khanam 2019; Oomah et al. 2002; Da Porto et al. 2012). The oil may then be further purified and refined.

Pressing is one of the oldest methods used for extraction of hemp oil, with seed pre-treatment and processing variables affecting the efficiency of oil extraction and quality of the oil. Seed pre-treatment (50°C/1 h), a higher temperature of extraction (70 °C compared to 50 °C) and slow rotational screw speed (22 rpm versus 32 rpm) increased the oil yield (Crimaldi et al. 2017). The conditions used for cold-pressing can also affect the content of phenols and polyphenols in hemp seed oil (Faugno et al. 2019). Enzyme-assisted cold pressing has been found to increase the oil extraction efficiency without compromising oil quality (Latif and Anwar 2009). A comparison of solvent extracted oil from untreated hemp seed or hemp seed that were previously subject to a microwave treatment showed that microwave treatment improved oil yield, carotenoid and tocopherol contents but did not alter fatty acid composition and increased the resistance to oxidation (Oomah et al. 2002). Extraction of oil from hemp seed using supercritical CO_2_, cold pressing or solvent extraction resulted in oils with similar fatty acid composition, however oil extracted using supercritical CO_2_ had higher tocopherol content, but reduced pigment compared to cold pressed oil. Solvent-extracted hemp seed oil did not contain tocopherols due to the high temperatures used during extraction and had intermediate levels of pigments compared to oils extracted by the other two methods (Aladić et al. 2015). Due to high levels of polyunsaturated fatty acids, hemp seed oil is very susceptible to oxidation with virgin oil being especially sensitive. This is due to high contents of chlorophyll that acts as a photosensitiser (Matthäus and Brühl 2008).

Oil was traditionally considered the more valuable component of oilseed processing and the pressed cake and meal were considered to be by-products, initially used in the animal feed industry. With the recognition that oilseeds were also good sources of protein, a range of protein concentrates and isolates have been produced from the solvent-extracted oil seed, the mechanically pressed cake or seed meal produced after solvent extraction of the pressed cake (Shen et al. 2021). There appears to be some confusion around the labelling of hemp seed cake and hemp seed meal in the literature and market as sometimes these terms are used interchangeably. However, to be consistent with other oilseed literature, hemp seed cake should refer to product obtained after mechanical pressing of oil while hemp seed meal is obtained after solvent extraction of the pressed cake. After expelling the oil using a screw press, the oil content in the residue obtained has 8.4–15.5% oil (Callaway 2004; House et al. 2010) while the oil content in the de-lipidated hemp meal after solvent extraction is expected to be much lower (~ 1% oil) (Potin and Saurel 2020).

Oil in oilseeds is present as oil bodies which are covered by oleosin proteins. A method for simultaneous aqueous separation of oleosomes and protein from oilseed has been reported. Using this process intact oleosomes are recovered and may be used as a natural oil-in-water emulsion with high physical and chemical stability (Ntone et al. 2020). Methods for non-destructively manufacturing oleosomes have been patented (Gray 2014). These methods are expected to be applicable to other oilseed including hemp.

### Protein extraction

Methods used for protein isolation from oilseeds (Arrutia et al. 2020) may be applied to isolation of hemp seed protein fractions from hemp seed (Potin and Saurel 2020; Pihlanto et al. 2020). Understanding the source used for protein extraction and the prehistory of the starting ingredient for protein extraction is important as pre-processing can impact the protein extraction efficiency and protein quality. As the protein was originally considered a secondary product, the traditional methods did not consider the functionality or quality of the protein and often the processing steps used were detrimental to protein quality.

### Sequential extraction of protein from hemp seed

Aqueous extraction is the most common method used for extraction of protein and has been applied to whole seed, cake and meal, usually after oil is expelled by pressing or solvent extracted (Shen et al. 2021; Pihlanto et al. 2020). Tang et al. (Tang et al. 2006) prepared hemp seed protein isolate (86.9% protein, wet basis) by alkaline extraction and isoelectric precipitation, starting from de-fatted hemp seed meal (50.2% protein) obtained after dehulling and defatting using supercritical CO_2_ at < 40 °C. Compared to soy protein isolate prepared using a similar process, the hemp seed isolate had superior amino acid composition although its techno-functional properties (solubility, emulsifying activity and water holding capacity) were inferior (Tang et al. 2006). Using a similar process to Tang et al. (Tang et al. 2006), hemp seed protein isolate (84.15% protein) was extracted from a commercial defatted hemp seed meal (44.32% protein) (Malomo et al. 2014). Some high molecular weight proteins were not extracted from the hemp seed meal when using alkaline extraction and the authors reported that the pH of minimum protein solubility was pH 3.0 for defatted hemp seed meal and pH 4.0 for the hemp seed protein isolate (Malomo et al. 2014). The authors also found that the emulsifying properties of these fractions were different and dependent on pH (Malomo et al. 2014). Alkaline extraction/isoelectric precipitation of defatted hemp seed meal (47.9% protein) produced a protein isolate (91.44% protein) with a lower protein content than that produced using micellisation (salt extraction followed by dialysis and ultrafiltration) (98.87% protein) (Hadnađev et al. 2018). The pH of minimum solubility was pH 5.0 for the alkali-extracted protein isolate while that for protein extracted using micellarisation was higher at pH 6.0. In addition, the alkali-extracted protein had a higher degree of denaturation and water holding capacity but the fat holding capacity was not significantly different (Hadnađev et al. 2018). These variations in functionality provide opportunities to match differently prepared isolates to a range of applications.

The presence of fat in the starting material for isolation of protein is generally regarded as undesirable and often solvent extracted material is used. A recent review suggests that total de-fatting using solvent may not be necessary for some applications of protein-enriched fractions (Carre 2021). A study on the extractability of hemp seed protein from un-defatted hemp seed press cake under different hydration conditions, pH, ionic strength and press-cake liquid ratio suggested that pH was the most influential factor determining protein recovery (Potin et al. 2019). These authors found that pH > 9.0 significantly enhanced protein yields but increasing pH increased pigmentation and suggested that further steps are required to purify the protein (Potin et al. 2019).

Recently, newer approaches are being explored to obtain both high quality oil and protein fractions from plant sources. Using a non-solvent process, a highly digestible rapeseed protein isolate (90% protein) which is soluble between pH 2.0 and pH 12.0 with desirable emulsifying and foaming properties for applications in a range of manufactured food was reported, although the details of the process were not disclosed (Dereuder 2019).

### Simultaneous aqueous extraction of an oleosome-protein extract

Simultaneous extraction of oleosomes (oil bodies) and protein from oil seed meals have the advantage of being a relatively mild process not requiring organic solvents, high temperature or extreme pH. These methods also do not affect the solubility of protein (Ntone et al. 2020). There is a report on the properties of a spray-dried commercial hemp seed protein concentrate derived from filtration of the heavy aqueous stream of a liquid–liquid phase separation process for hemp oil body extract. It was suggested that a functional hemp protein concentrate (71% protein, 32.5% fat, 2.8% ash) with an isoelectric point of pH 5.2–5.3 was obtained. The hemp protein concentrate isolated from full-fat hemp was a good foam stabiliser (Galves et al. 2019). A recent research study examined the recovery of hemp oleosomes from hemp milk. The hemp milk was produced by separation of a previously colloid-milled dehulled hempseed/water mixture using a clarifier separator and hemp oil bodies were separated from the hemp milk by centrifugation (Garcia et al. 2021).

### Extraction of phytochemicals

Other bioactive components may be extracted from de-fatted hemp seed cake and serve as extracts for preparation of functional foods with health promoting properties (Rupasinghe et al. 2020). A comparison of conventional extraction of hemp seed cake with a mixed solvent with ultrasound-assisted extraction showed that higher amounts of polyphenols and flavonoids are extracted with the application of ultrasound and further that the antioxidant capacity of the extracts were also increased (Teh and Birch 2014).

## Applications in food

There is significant potential of hemp-based ingredients to be used in a range of food applications (Leonard et al. 2020; Rupasinghe et al. 2020; Xu et al. 2021). However, there is also emerging interest in the use of other parts of the plant (e.g. sprouts; leaves; sap extract from leaves). The use of hemp ingredients in food is subject to the ingredients having low levels of THC and CBD. There are different limits for these components in hemp food products in various countries and jurisdictions. As an example, the permissible levels allowed in Australia, the EU and the USA for various hemp products (Government of South Australia 2020) are given in Table 1.

**Table 1:** Maximum permissible THC contents in different hemp food products in Australia/New Zealand, the European Union and the USA

Hemp product	Australia/New Zealand^**a**^	European Union^**b**^	USA^**c**^
Industrial hemp plant	0.35% or 1% dw	0.2% dw	0.3% dw
Hulled hemp seed	5 mg/kg	3 mg/kg	4 mg/kg
Hemp seed oil	10 mg/kg	7.5 mg/kg	10 mg/kg
Hemp flour and hemp protein powder	10 mg/kg	3 mg/kg	Not specified
Hemp seed milk or other beverages	0.2 mg/kg	Not specified	Not specified
Milled hemp seed as ingredients	5 mg/kg	3 mg/kg	Not specified

^a^Food Standards Australia and New Zealand specify limits for *Cannabis sativa* seeds as food and ingredients in foods. FSANZ state that industrial hemp for food is limited at 1% THC in leaves and flowering heads, though some states have set lower limits of 0.35%. Australia also specifies that hemp seed products must contain no more than 75 mg/kg of CBD

^b^The European Commission amended Regulation (EC) No. 1881/2006 to set maximum limits for hemp foods in Europe, though each state is free to set their own limits equal to or below these limits. The amended regulation takes effect 1 January 2023

^c^The US Food and Drug Administration has not set formal limits for THC in hemp-based foods. Though the “Generally Recognised as Safe” classification from the FDA specifies contaminant limits including THC which are presented

Table 2 provides examples of selected applications of hemp seed products in formulated foods. Overall, hemp seed flour has superior nutritional quality compared to wheat flour and may be used for fortifying food products, as it is higher in protein, fat, minerals, fibre, essential amino acids and essential fatty acids compared to wheat flour (Rusu et al. 2021). The high level of polyunsaturated fatty acids in hemp seed oil means that it may be used as a nutritious oil for salad dressings but is it is not suitable for deep frying due to the high level of unsaturation in the oil. Proteins from oilseeds like hemp are seen as alternative protein sources to animal protein, contributing to the production of acceptable meat analogues (Zahari et al. 2020), and a acting as a valuable source of nutritional and bioactive components (Arrutia et al. 2020; Kotecka-Majchrzak et al. 2020).

**Table 2:** Applications of hemp seed products in food

Application	Functionality in food	Reference
**Bakery products**		
*Cookies*: substitution of wheat flour (up to 20%) with hemp flour (raw or roasted)	Hemp flour containing cookies had higher ash, protein, phenolic content and anti-oxidant activity but were softer	(Ertaş and Aslan 2020)
*Starch based gluten free bread*: partial substitution of starch in recipe with flour or protein-enriched fractions	Part substitution of starch with hemp flour weakened dough structure, while incorporation of hemp protein concentrate (20%) reinforced dough structure. Both incorporation of hemp flour/hemp protein concentrate improved nutritional value and sensory properties	(Korus et al. 2017)
*Bread:* partial substitution of wheat flour (up to 80%) with hemp seed flour made from pressed hemp seed cake	Bread with hemp seed flour had higher nutritional value; dough stability and strength was not affected by up to 10% substitution of hemp flour	(Pojić et al. 2015)
**Beverages**		
*Fermented drinks*: hemp drink (containing 3% hemp seed) or 1:1 (v/v) mixtures of hemp-soy or hemp-rice drinks fermented with probiotics	Hemp seed fermented drinks had strong prebiotic activity; basis for producing prebiotic and probiotic plant-based drinks for the alternative dairy market by fermentation	(Nissen et al. 2020)
*Hemp seed milk beverage*: non-thermal processed hemp milk (4% protein, 5% fat)	High pressure homogenisation (up to 60 MPa) in combination with pH shift (to pH 12) may be used to produce physically and oxidatively stable milk	(Wang et al. 2018)
**Confectionery**		
*Extruded energy bars*: partial substitution of rice flour (up to 40%) with defatted or whole hemp powder	Bulk density increased while equilibrium moisture content decreased with hemp powder incorporation; bar made with extruded rice/20% whole hemp had higher overall sensory acceptability	(Norajit et al. 2011)
**Plant-based meat analogues**		
*High moisture extrusion formulations:* partial substitution of soy protein isolate with hemp protein concentrate in meat analogue formulations	Extrusion in twin screw co-rotating extruder was possible with up to 60% wt. replacement of soy protein isolate and resulted in a meat analogue with a comparable texture to soy protein isolate alone	(Zahari et al. 2020)

Hemp seed products are emerging ingredients in the food industry. With this comes the challenges of development of ingredients with consistent composition and techno-functionality. Starting with the application in mind and understanding functionality in the end application will help in the design of fit-for-purpose ingredients through appropriate breeding technologies, post-harvest handling practices and processing operations to develop sought-after value-added ingredients for the food industry.

## Recommendations

A successful and profitable industrial hemp supply has to be built to addresses a market-led opportunity. Solutions to overcome current barriers and enable the supply chain to flourish requires identification of a whole-of-supply chain approach with multiple stakeholders. For the food industry, consumer acceptance of hemp food ingredients and products will be of vital importance. Some of the issues that need further attention, to add to the growing body of knowledge in the industrial hemp industry, include the following:

On farm:

- Reliable seed supply of stable and genetically defined cultivars
- Breeding strategies to enable production of hemp crops with low THC under stressed conditions (e.g. understanding gene expression for cannabinoids under hot conditions and in drought)
- Breeding (new cultivars)/farm management practices (digital agriculture) to reduce water requirements whilst maintaining desired farm productivity
- Robust processes (e.g. safe and reliable feminisation protocols) for consistent seed supply
- Mechanical harvesting processes for improved efficiency

Post-harvest:

- Safe storage and transport conditions for maintaining hemp seed quality
- Food safety protocols and pest-control measures

Processing of hemp seed:

- Pre-conditioning of seeds for improved processability
- More sustainable methods for separation and processing of hemp seed
- Improved processing protocols for obtaining protein fractions of consistent quality
- Novel methods to protect hemp seed oil against oxidation (e.g. encapsulation)
- Developing processes for separation of protein and intact oleosomes
- Systematic characterisation of inherent nutritional and functional properties of hemp seed flour, protein fractions and hemp seed oil obtained using various processes

Food applications:

- Developing ingredients with fit-for-purpose functionality in end applications
- Testing applications of hemp seed protein in traditional food applications (e.g. bakery, snacks) and in new emerging markets (e.g. meat analogues, alternative dairy)

Consumers:

- Communication to improve consumer understanding (e.g. difference between *Cannabis* for recreational drug use and industrial hemp with low THC for food use)
- Education about the nutritional properties of hemp seed ingredients and products
- Development of recipes containing hemp ingredients

Market/Trade:

- Harmonised specifications for trade of hemp seed ingredients (i.e. hemp seed, hemp protein, hemp seed oil)
- Standardised methodology for testing and analysis of THC in food products
- Understanding of heterogenous regulations for cannabinoids in hemp food products in various jurisdictions

## Conclusions

The growing of industrial hemp, containing low levels of THC, to provide food ingredients and incorporation into finished products is a significant opportunity. The market-led opportunities hold promise for hemp seed, which was traditionally regarded as a by-product of the hemp fibre industry. Hemp seed is a valuable product in its own right for the food industry, which can be further developed into value-added ingredients. The excellent attributes of hemp seed are their high nutritional value (high in nutritious protein and oils) and the techno-functionality of the hemp protein for a world that is short of protein. The ability to increase the productivity of the hemp crop with consistently low THC content will be a pre-requisite for investment in the industrial hemp industry as farmers need assurance that their crops can be harvested for the use they were grown for. Overall, the opportunity to build a whole supply chain is particularly attractive for the farm sector wishing to diversify and plant sustainable crops with potential for economic returns.

## Data Availability

No new data or materials were generated in this manuscript.

## Abbreviations

CBD: (-)-cannabidiol
cv.: Cultivar
kDa: Kilodalton
MW: Molecular weight
THC: (–)-*trans*-Δ^9^-tetrahydrocannabinol
USD: United States Dollar
